# Rethinking Economic Measurement Using Statistical Ensembles

**DOI:** 10.3390/e27030265

**Published:** 2025-03-03

**Authors:** Cal Abel

**Affiliations:** Signal Power and Light, Inc., Cordova, AL 35550, USA; crabel@signalpowerandlight.com; Tel.: +1-205-536-9200

**Keywords:** Allais paradox, expected utility, production function, income distribution, income inequality, entropy, utility, C01, C51, C70, D01, D63, D81, E01, E17

## Abstract

The axiomatic framework of quantum game theory gives us a new platform for exploring economics by resolving the foundational problems that have long plagued the expected utility hypothesis. This platform gives us a previously unrecognized tool in economics, the statistical ensemble, which we apply across three distinct economic spheres. We examine choice under uncertainty and find that the Allais paradox disappears. For over seventy years, this paradox has acted as a barrier to investigating human choice by masking actual choice heuristics. We discover a powerful connection between the canonical ensemble and neoclassical economics and demonstrate this connection’s predictive capability by examining income distributions in the United States over 24 years. This model is an astonishingly accurate predictor of economic behavior, using just the income distribution and the total exergy input into the economy. Finally, we examine the ideas of equality of outcome versus equality of opportunity. We show how to formally consider equality of outcome as a Bose–Einstein condensate and how its achievement leads to a corresponding collapse in economic activity. We call this new platform ‘statistical economics’ due to its reliance on statistical ensembles.

## 1. Introduction

Recent work reformulating the axiomatic foundation of game theory [1] gives us a new footing for exploring economics. This work drastically restructures our economic thinking, forcing us to discard entire fields of study as being irrelevant or in direct opposition to a fundamental law of nature, the second law of thermodynamics. Moreover, it shows that we have not been using the correct metrics. While this approach discards many things, it strengthens some familiar conclusions. It does so by giving them a new, more meaningful context and significance, such as formally connecting neoclassical economics to game theory.

Our issue is not the data or tools we do not have. All of the topics in this paper could have been developed under the existing neoclassical and game theoretic frameworks. The data that we employ in our analysis are well-studied and by no means novel. The difference is in how we apply our tools and interpret the data.

The only new concept that we introduce to an economist is that of the statistical ensemble. It is not that this concept was not available; it was, but we did not recognize it. All it took was generalizing game theory and its axioms to lift the scales. Welcome to statistical economics.

### 1.1. Isomorphisms

The cross-pollination of ideas in economics and the physical sciences has a rich and significant history. This history is a testament to the shared goal of understanding both the worlds of people and things. This concurrent development comes from sharing the same fundamental mathematical structures, isomorphisms, which differ in only superficial properties [2].

Game theory’s new axiomatic basis [1] leverages such an isomorphism. In the 1960s, Pfanzagl [3] developed an axiomatic theory of measurement, a framework that provides a systematic way to assign numbers to objects, cardinality. Pfanzagl used this theory to show the equivalence of Savage’s subjective expected utility to von Neumann–Morgenstern utility. When we look at the axiomatic formulation of quantum mechanics, we see that quantum theory is fundamentally a theory of measurement.

Following Pfanzagl’s methods, QGT proved the functional equivalence between von Neumann entropy and vNM utility [1]. This work follows a long-established trend outside of mainstream economics, attempting to identify entropy’s role in economics (Jakimowicz [2] presents a meticulous summary of the historical prior work.) This effort is motivated by the second law’s attractiveness as an absolute law shaping dynamical behavior.

While an economic entropic isomorphism is clearly needed, it must be precise because there are many different entropies. There is only one second law and only one associated entropy with that law: the ensemble’s entropy (the ensemble entropy is the von Neumann entropy in the quantum context and the Gibbs entropy classically).

Previous applications of entropy in economics did not make this subtle and essential distinction, rendering them fundamentally flawed. Economists’ reluctance to apply isomorphisms without a proper theoretical basis is not incorrect, as prior entropy theories led to erroneous conclusions.

However, this hesitance is not entirely justified either; historical isomorphisms, such as production functions, applied out of empirical necessity were not only insightful but also later explained theoretically. This experience validates the physical science motif of observation first, followed by advancing theory. Experiential primacy is also true neurobiologically [4]; it behooves us not to place the cart before the horse.

### 1.2. Background of Ensembles in Economics

The formal application of ensembles in econophysics is a relatively recent advancement [5]; however, their application in mainstream economics remains limited due to the absence of an axiomatic justification [2]. Ensembles, or rather some of their metrics, have appeared in the economic literature [6,7], but these have not had any significant development.

Econophysicists seem content with developing isomorphisms, while economists are equally content to ignore these developments [8]. Until QGT proved utility to be the ensemble’s entropy [1], there was no theoretical framework to overcome this impasse. This proof revealed that many had mistakenly identified utility as being isomorphic with energy (This misconception began with Irving Fisher and was cited and even propagated by Jakimowicz [2]. I also held the same misconception until the proof was complete, and even then, I was still skeptical).

This misidentification limited economists’ and econophysicists’ ability to identify and adequately conceptually frame utility into a meaningful metric. Furthermore, entropy’s historical characterization of disorder is not helpful. A better conception of entropy is as a measure of complexity or, as we will find out shortly, potential.

We need to consider both open and closed systems because the behavior in each is opposite regarding the minimization or maximization of entropy, respectively. We can think of the closed thermodynamic system as the world around us or as a competitive game. It is a world where entropy is *always* maximized. This entropy maximization is the second law of thermodynamics; it is inexorable and unavoidable. An open system extracts the potential of the world around us and creates pockets of lower entropy through work expenditure, which increases global entropy (The behavior of an open system is the same as Maxwell’s daemon. Szilard presented a mechanism of how this daemon acts to increase global entropy, preserving the second law [9]). The open thermodynamic system describes the fundamental aspect of life and, by extension, economics.

Life transforms the chaotic potential of the world into low-entropy things. We must work to create and maintain these pockets of lower entropy (Work, as referred to here, is, by definition, the expenditure of exergy). This conception of entropy is directly contrary to the formulation of entropy economics by Georgescu-Roegen, which postulated that the action of human beings was to take low entropy resources from the environment and transform them into high entropy activities [2]. As a direct result, entropy economics suggests that there are material limits to growth. What we are presenting is that the limits to growth are solely dependent upon our use of energy to maintain the desired state of the world we create.

In recorded history, this pattern of behavior has played out time and time again: the Industrial Revolution, where we harnessed energy long stored in the ground and created means of converting it into a mechanism capable of supporting a population far more extensive than the conception or even belief of Malthus and our increasing ability to extract order from the potential around us, which is why Paul Erlich lost his bet with Julian Simon. Open systems must create and exploit an entropy gradient in order to perpetuate themselves.

If these systems allow themselves to reintegrate with the increasing entropy of chaos, we have, by definition, their death and dissolution. Our open economic ensemble or any other open ensemble (e.g., species) will reach a limit defined by its ability to access energy. Unfortunately, economists have not recognized and/or refused to acknowledge the centrality that energy plays in our lives and life in general [10]. The application of energy in society determines the “extent of the market” ([11] Book 1, Ch. 3), and the measured entropy quantifies the “division of labor”.

The pattern of reducing entropy and then increasing entropy by adding energy is essential for the function of any heat engine. In the economic context, we, as individual participants, work to reduce the potential of the world around us and, through the addition of energy, increase the complexity of our ensemble/society.

We witness this correctness even beyond empirical and theoretical framing. We see it in our societies’ oldest stories, such as the Enūma Eliš or the Book of Genesis. In both stories, the Creator makes Creation out of the chaos of the primordial soup, converting the potential of the world into something structured: in this case, life ([12] pp. 2–3). Genesis goes a step further; God enjoins a member of His Creation to name everything and assert dominion and care, analogous to God’s act of Creation. We can interpret this as God tasking humanity with the stewardship of life and an invitation/commandment to emulate His work of transforming the potential into the useful ([12] p. 7).

Touching on religion is unavoidable because we, as human beings, base our actions and choices on value (The canonical distribution, Equation (3), shows this concept of value-directing action through the Hamiltonian, $\hat{H}$, determining choice, $\hat{\rho }$). Language even provides a value structure, restricting and directing our focus from the innumerable chaos of potential to a set of “things” with which we can use ([12] pp. 13–17). We see the world through a story, and the story we choose defines the world and our possible interactions ([12] p. 11). This use of the word value simultaneously contains the numeric representation and the ineffable subjective. What is “good”? Genesis outlines a framework where the continued act of Creation and the continuation of life are“very good”.

So, what direction do we point to as being where we aim in policy and philosophy? The choice is stark; we can choose life or death. Humans can be considered either a flawed embodiment of the good or a cancer on the planet that must be eliminated because of their consumptive tendencies. However, if we take a step back, all life is consumptive; thus, all of life and Creation, in that mindset, should be destroyed. Again, what is “good”?

### 1.3. What Changed to Now Allow Using Ensembles?

The fingerprints of ensembles have always been with us. Only the current development of econophysics leads the effort to apply ensembles empirically in economics [2,5]. It is interesting to note that it is entirely possible to develop and apply economic ensembles using a purely classical approach, just like Gibbs did in physics. However, economists did not develop ensembles; they practically ignored expected utility, which was a necessary tool in their development.

In physics, ensembles manifest an extensive property known as entropy, *S*, which, as Boltzmann pointed out, is closely related to the number of possible arrangements or microstates of a system that result in the same macroscopic properties (multiplicity), *W* (If k=1, entropy is dimensionless in its natural unit).

(1) S=klogW.

However, multiplicity is never really considered in economics. Daniel Bernoulli mentioned it about 150 years before Boltzmann [6], but it was never rigorously pursued. Jakimowicz’s extensive review of entropy in economics neither mentions multiplicity nor ensemble entropy [2]. It presents some adjacent notions but does not fully delineate them. This paucity of literature is a significant indication that all previous expositions of entropy in economics do not use the ensemble’s entropy, confusing any subsequent interpretations.

vNM utility presented an avenue to develop the concept of ensembles formally. However, economists effectively ignored/limited the application of classical game theory because of the perceived flaws in the vNM formulation of utility. Morgenstern even noted that economists such as Allais were being short-sighted and that a different axiomatic formulation would render Allais’s and others’ arguments moot [13].

The axiomatic formulation of QGT, the proof of classical game theory as a special case of QGT, and the proof of the equivalence of von Neumann entropy and vNM utility, provided the necessary and sufficient foundation to discard the earlier critiques of game theory and EU [1]. By removing the independence axiom as a foundation of game theory, the Allais paradox has nothing to contradict. Because this paradox is so famous, we will use it as an example of how to show earlier critiques of game theory as irrelevant.

## 2. Materials and Methods

### 2.1. What Is an Ensemble?

We are using the word ensemble in what seems like a vague “suitcase” word that has no specific meaning because it can mean anything. This vagueness is due to how we measure/define the ensemble, which determines its behavior. The definition of an ensemble is, however, straightforward; an ensemble is a collection of things on which we can make some set of observations. In the quantum context, the ensemble average of an observable is the trace of the product of the potential observable outcomes, $\hat{M}$, multiplied by the probability observable, known as the density matrix, $\hat{\rho }$ (See reference [1] (Def. 2.9) for the formal definition of a density matrix).

(2) $$\langle M\rangle =\mathrm{error}\hat{\rho }\hat{M}.$$

These observables that scale with the ensemble’s size are called extensive (Some observables do not scale with the size of the ensemble. Price is an example of such an observable. This class of observable is called intensive). In the classical context, we observe some distribution of a quantity and then compute the average of that observation.

Some examples of ensembles are those of an individual across time or of a group of individuals at a specific time. Time and value are complementary measures in economics, as are their isomorphs, time and energy, in physics. Due to their complementarity, they obey the uncertainty principle. If we are to know something’s value, we cannot place the time; if we know the specific time, we cannot identify the value.

Some will react that this is an entirely subjective framework. It is; we can only measure and observe in finite time. As individuals, we cannot exist as each possible incarnation of the potential that we embody. We exist in finite time and finite locality. However, when we look at even just moderately sized populations, the ensemble behaves as if it is composed of indistinguishable entities behaving ergodically. For this reason, the assumption of ergodicity has some interesting consequences that may make those of a frequentist persuasion subjectively Bayesian; we as individuals are acting as if extensions of some quantity of “humanness” that is indistinguishable from one another and indistinguishable from other people across time. This interpretation will undoubtedly result in some interesting debate.

One of the more important properties of an ensemble is its distribution, which is straightforwardly derived from QGT’s axioms [1],

(3) $$\hat{\rho }=\frac{e^{-\beta \hat{H}}}{Z[\beta ]}.$$

The term $\hat{H}$ represents the observable of an individual’s or group’s values. The term β is a Lagrangian multiplier used to maximize the ensemble’s entropy/utility given the Hamiltonian. In a game theory setting, β represents a measure of the risk preference of the individual. We generally usually use its inverse, *T*, which has units of value (energy) and represents a measure of the economic activity of the ensemble. The term $Z\left[\beta \right]$ is the partition function, the normalization constant, and is a pure functional of β (The partition function is very important; its logarithm represents a thermodynamic potential, but we restrict its use here, as this discussion would expand the scope of this paper. An interested reader should look at [1] (§ 2.e(iii)) or [14] for more detail).

There are many paths to deriving Equation (3), but the most clear and direct is through the integration of the axiom relating the time evolution of states with the Hamiltonian; if we add up the sum of the small choices based on our values, we arrive at the distribution of our actions as individuals and as groups of individuals. This concept also intuitively explains the uncertainty principle and the complementarity of time and value.

We can express different distributions of different ensembles, but that is not necessary for what we need here, although the concept is similar. The eigenvalues of the density matrix are the probabilities of classical game theory and, strictly speaking, represent our observations.

As Jaynes notes, the canonical distribution is a maximum entropy distribution [14]. Equation (3) represents any possible distribution (determined by the Hamiltonian), finite or continuous. Matsoukas had previously generalized and extended this concept classically [15].

### 2.2. The Distinction Between Classical and Quantum

One of the isomorphisms that quantum mechanics borrowed from economics is that the collapse of the wave function can be thought of as if the quantum object “decides” to be in one state or another based on the Hamiltonian. The quantum superposition represents an “undecided” state before a choice. This is a difficult anthropomorphism to ascribe to perceptibly inanimate objects. However, when we apply this analogy to clearly thinking and reasoning entities, we clearly distinguish between the interpretation of the quantum and classical.

The quantum state represents the undecided state, where we hold each of the possibilities of an outcome in a superimposed state. When we make a decision and subsequently demonstrate, make observable, that decision through action, we provide direct, measurable evidence of the collapsed choice. We can see why Rothbard insisted so stringently on the primacy of demonstrated preference.

This restriction severely limits us as experimentalists. We can only observe what we can measure; we only know the people we watch by the fruits of their choices. Thus, just like in quantum mechanics, we can only infer the structure of the Hamiltonian, the values of a person. Their entanglements influence their decisions and, thus, correspondingly, what we observe.

A way of thinking about entanglements is as relationships developed through repeated past interactions. One example of this would be a husband and a wife acting predictably as individuals with shared values of the couple. The Tit-for-Tat solution to the prisoner’s dilemma is another example of how such entanglements can form in competitive games. In a business setting, a contract represents an agreed-upon structure of entanglement that excludes Nash equilibria to hopefully some *de minimus* probability.

In a society, evolved norms provide predictability to the behavior of its members and a means of identifying the group as a whole from other groups that have a different set of values. Suppose we mix two ensembles with drastically different values. In that case, they are either compatible and work in concert or incompatible, where one takes advantage of the other based on the differences in the value operators.

### 2.3. Thermodynamics

The direct consequence of using an ensemble approach is that we describe the time evolution of the ensemble’s distribution through a set of differential equations or directly through the Euler equation. This methodology is no different than that of neoclassical economics. What is made explicit is that these representations describe a distribution of the observed parameter and that without the inclusion of the distribution’s entropy, those relationships are fundamentally incomplete.

There is no fundamental difference in the equations that we derive from an ensemble compared to those long familiar with neoclassical economics. What is different is what we are measuring and the explicit inclusion of the ensemble’s entropy.

In mainstream economics, the concern is over attempting to describe an individual’s utility function. Utility, while maximized, is not what motivates people; their values motivate them. Entropy/utility maximization drives the process; it is the “Invisible Hand” (Yakovenko illustrates *exactly* the process of entropy maximization that Smith is describing as the function of the invisible hand [5] (III.C)):

They are led by an Invisible Hand to make nearly the same distribution of the necessaries of life, which would have been made, had the earth been divided into equal portions among all its inhabitants [16].

(Part 4, Ch. 1)

Furthermore, the open system maximization of each individual’s values leads to the wealth of nations.

By preferring the support of domestic to that of foreign industry, he intends only his own security; and by directing that industry in such a manner as its produce may be of the greatest value, he intends only his own gain, and he is in this, as in many other cases, led by an Invisible Hand to promote an end which was no part of his intention [11].

(Book 4, Ch. 2)

We have known these phenomena for a long time but have been unaware of the underlying mathematical structure. Value, not utility, motivates choice; utility is the exploratory force that drives choice. Thus, our primary concern is attempting, however imperfectly, to discern others’ values, their Hamiltonian.

## 3. Results

Now that we have our tools, we can start to work on their application.

### 3.1. The Allais Paradox

List and Haigh conducted an interesting experiment looking at how professional risk takers, traders on the Chicago Board of Trade, evaluated risk compared to that of a control cohort of college students [17]. This difference in experience is a fortunate distinction for us to consider because we can evaluate the ability of each cohort to discern risk and select the optimal (for them) outcome. In the simple games, the students and traders behaved consistently, following nearly identical strategies. There was a difference in risk evaluation in the more complex composite game. The traders correctly identified the joint game as a composite game, whereas the students naively assumed that the games were independent.

We use the conventional ket, |i〉 (Paul Dirac developed a compact and powerful notation to express the vectors and operators in quantum theory. Readers should familiarize themselves with their application, as we will be using Dirac’s notation throughout this paper.), with a numeral, *i*, to represent each orthonormal choice, called a basis, that List and Haigh presented to the students and traders:

- |1〉 Win $7 with certainty.
- |2〉 Win $7 with 75% chance, $10 with 20% chance, and $0 with 5% chance.
- |3〉 Win $7 with 25% chance and $0 with 75% chance.
- |4〉 Win $10 with 20% chance and $0 with 80% chance.

The first game, *A*, was the choice between |1〉 and |2〉. The second game, *B*, was the choice between |3〉 and |4〉. There was a final compound game, AB, of having to choose one outcome from each game and be paid the outcome of a sub-lottery between those two games with even probability. The compound game had the following choice sets |1,3〉, |1,4〉, |2,3〉, and |2,4〉.

We assume the choices of each game are independent of each other. Thus, the payout matrix, Hamiltonian for each game is ${\hat{H}}_{A},{\hat{H}}_{B},{\hat{H}}_{AB}$ for the first, second, and joint games, respectively:

(4) $${\hat{H}}_{A}=\left[\begin{array}{cc} 7 & \\ \; & 7.25 \end{array}\right],\;{\hat{H}}_{B}=\left[\begin{array}{cc} 1.75 & \\ \; & 2 \end{array}\right],\;\mathrm{and}\;{\hat{H}}_{AB}=\left[\begin{array}{cccc} 4.375 & \; & & \\ \; & 4.5 & \; & \\ \; & \; & 4.5 & \\ \; & \; & & 4.625 \end{array}\right].$$

The observed data for the outcomes of each of the games for each cohort are in Table 1.

Using the Hamiltonians, the empirical density matrices, and the number of players *N*, we find the empirical entropies 〈s〉, expected payout 〈e〉, and through a simple regression β for each game as in Table 2 (We are following the convention of the total entropy as being 〈S〉=N〈s〉, where 〈s〉 is the specific entropy of the individual; similarly, we have 〈E〉=N〈e〉. Also, note that the units of β are the inverse units of the expected payout).

The “Canonical” for game AB in Table 2 represents the situation where the traders acted optimally for their estimated risk preference, β. The absence of the β for the students is that their strategy resulted in a much more complex Hamiltonian than can be expressed here. The student strategy in the compound game was

(5) $${\hat{\rho }}_{AB}={\hat{\rho }}_{A}⊗{\hat{\rho }}_{B}$$

with a goodness of fit of $\chi^{2}\left(3,\;N=30\right)=1.20,\;p=0.78$ using the empirical density matrices of the first cohort of students. This model suggests that the students treated the games as being distinct and not as an integrated composite. Furthermore, we can also conclude that the traders, who evaluate risk professionally, were able to consider the composite game as a composite and get close to an optimal outcome for their observed risk preference.

If we wanted, we could investigate why people prefer the “sure thing” slightly more than the optimal. This sure thing preference is significantly more telling for the students than the traders, but the effect clearly remains for traders, even showing up in the composite game. More work is needed to explore the Hamiltonians’ off-diagonal components and explain the “sure thing” preference. The preference of the “sure thing” also appears in neuroscience, where people act to limit choice (entropy) to be settled and that too much choice can induce anxiety [18].

Because we are dealing with a single group of people for each cohort, their risk preference should be the same from game to game. Because it is not, we have some other factor, likely the “sure thing”, modifying their preference. It is interesting to note that the risk preferences for the traders for Game *B* and AB are very close, giving credence to this line of thought.

We clearly do not have a paradox when using statistical economics. Instead, we uncovered that

1. Novices use simple heuristics when confronted with complex games.
2. People have a quantifiable preference for the “sure thing”.

How long have we been ignorant of being able to quantify these effects, and to what other insights have we made ourselves blind due to a frivolous and pedantic argument?

### 3.2. Econometric Analysis

Next, we turn to applying our theory to the macro scale and look at large populations outside of carefully constructed experiments, people acting in the wild, so to speak. Before we begin, let us take a step back and frame what we are going to be doing in the context of existing economic thought. As we noted earlier, Daniel Bernoulli [6] long ago proposed that the utility functional was logarithmic. In macroeconomics, there is a power law relationship known as the Cobb–Douglass production function,

(6) $$Y=A\;L^{\beta }K^{\alpha }.$$

where *Y* is the total economic output, *A* is the total factor productivity, also referred to as the Solow residual, *L* is labor input, *K* is the capital input, and α and β are Lagrangian multipliers. The connection between the Cobb–Douglas production function and Bernoulli’s utility is that they both consider total productivity as being proportional to utility.

In physics, we see a very similar equation as the fundamental equation of state describing an ideal gas [19] (§3-4). This connection is beyond mere coincidence. An ideal gas and people share the same underlying stochastic process; they are canonically distributed, as in Equation (3). The continuous distribution with a finite mean and the highest entropy is the exponential distribution, which is canonical. Energy in an ideal gas is distributed exponentially amongst the component atoms, just like income is distributed amongst people [20]. The canonical distribution of income does not just occur in the United States; it also holds, when studied, in 67 other countries [21]. Thus, it is clear that the correct interpretation of income distributions is as statistical ensembles.

#### 3.2.1. Data

The next task is to determine which parameters to use in our model. We begin with the income data from the Internal Revenue Service from the 24-year period 1996–2019, as reported in Publication 1304, Table 1.1 [22]. The IRS data directly give us the total income and the number of taxpayers (There are a number of different metrics for income. We will use Adjusted Gross Income as our measure of income. AGI allows us to isolate the business activity of Limited Liability Companies, partnerships, or privately held companies with income reported on an individual’s K-1. The residual profit from these activities will be considered residual income of ownership, which AGI represents). The income distribution has income bins of different income levels over the years. To estimate the income distribution f(m), we will use the mixed exponential–Pareto distribution of Banerjee and Yakovenko [20],

(7) $$f\left(m;T^{⋆},m_{0},\alpha \right)=\frac{e^{-\frac{m_{0}}{T^{⋆}}\arctan\frac{m}{m_{0}}}}{Z{\left(1+{\left(\frac{m}{m_{0}}\right)}^{2}\right)}^{\alpha }}.$$

Equation (7) is then integrated over the bin width to estimate the hyper-parameters: $T^{⋆}$—the monetary temperature of the thermal portion, *Z*—the partition function, $m_{0}$—the thermal–epithermal crossover income, and α—the Pareto exponent using HMC with NUTS in Stan in the R statistical analysis framework. Since this is a relatively straightforward parameter estimation and the results differ little from previous work [20], we will not cover the specifics of the model or its development. The hyperparameters of Equation (7) are in Table A1. The ensemble’s entropy is then computed numerically for each year’s income distribution.

Ayers and Warr performed some extensive econometric work studying the impact of energy on the economy, specifically the Solow residual [10]. They showed that the Solow residual (which represents roughly 80% of GDP growth) could be explained entirely by the exergetic input into the economy (Exergy is a technical term that describes useful work extracted from a heat engine. Since all of our work comes from heat engines, exergy represents the measure of work done in/by the economy). Their work seems to have fallen on deaf ears in the mainstream economic community, as economists still treat energy as just another commodity instead of being central to economic activity [10] (p. 224). The entirely predictable economic collapse of Germany due to *Energiewende* is directly attributable to their artificial restrictions on energy supply. As Ayers and Warr note, making energy expensive reduces energy consumption, and all goods and services that depend on energy are reduced [10] (pp. xxi, 217–218).

To obtain the needed data to estimate the total exergy consumed each year, we used the Energy Information Agency’s Open Data API [23]. The data sets used in the analysis were the total energy consumed in a given year for the Residential (TERCBUS), Industrial (TEICBUS), Commercial (TECCBUS), and Transportation (TEACBUS) sectors. We multiplied the sectors’ consumed energy by the sector’s conversion efficiency: 65%, 49%, 65%, and 21%, respectively, to determine the exergy consumed by each sector [24].

#### 3.2.2. Model

The extensive parameters for the ensemble of tax-paying individuals are the size of the ensemble *N* from the total number of taxpayers, the total income *M*, the ensemble’s specific entropy *s*, the partition function *Z*, and *E*, the total exergy input into the economy. It is paramount for us to understand the metrics we have at our disposal, as our understanding determines how we interpret them and their implications. By employing analogs to the equations of state for an ideal gas, we create a clear connection between income distributions and thermodynamic concepts. It is worth noting that while some of these relationships may be familiar to an economist, the novelty lies in their derivations.

The first hurdle we must overcome is grasping how to view the income distribution, Equation (7), and understanding its representation in the thermodynamic concept. Taking our income distribution, our first estimation of its Euler equation is (The use of the word estimation is because the selection functional (see [15] (§3.8.1)) was not formally determined; thus, the terms in Equation (8) do not have an exact correspondence to those in Equation (7). They have a conceptual equivalence but are not mathematically precise.),

(8) $$S=\beta_{M}M+N\log Z.$$

The physics envy that economists have undeniably had with such accusations of “determinism” undoubtedly stems from a lack of understanding of what the physical sciences do. Equation (8) does not represent a physical process per se; it represents, *completely*, the distribution of some measurable ensemble quantity. Equation (8) is an equivalent mathematical representation of the observed income distribution. Thermodynamics, entropy, and the second law are not physical quantities, per se; they are stochastic properties that have physical manifestations and consequences.

Because we have not formally determined the selection functional for Equation (7), $T^{⋆}\neq T_{m}$, where from Equation (8), $\beta_{m}\equiv 1/T_{m}$. While not precisely matching the actual partition function, we will take the regressor *Z* from Equation (7) as being sufficiently representative of the actual partition function. We can then rearrange Equation (8) as

(9) $$T_{m}=\frac{m}{s-\log Z}.$$

where *m* is the average income and *s* is the numerically estimated Shannon entropy of Equation (7) and Table A1.

Next, we need to consider the exergy available to the ensemble. Because exergy is closely related to energy, we will place it in the same context as energy in statistical physics. This connection gives us an isomorphism between value and exergy. Because we must expend exergy and time in everything that we do, the measure of the time and exergy that we spend gives us a measure of our value of anything. Here, we present a purely subjective argument of value, but one that is balanced by an objective measure. For this reason, we consider our ensemble a single-commodity economy, money, where the total activity in the economy is given by exergy. Let us think about the model more conventionally. In this case, the exponential part of the distribution represents the payments to Labor, *L* of the Cobb–Douglas production function. The Pareto portion similarly represents the payments to Capital, *K*. These traditional economic components are still in the model but are incorporated drastically differently into the income distribution.

There is one item left to consider: What is the thermodynamic analog to money? Money is analogous to volume in thermodynamics. Money is the space in which economic activity occurs. We make the distinction here that it is not the total money supply that matters but rather the money available to an individual from their income. People ultimately own all companies. Thus, the individual realizes any profit that accrues to the company as income. As a result, we ignore companies’ paper value (stock price) and only consider them based on their dividends (realized gains).

Furthermore, we ignore individuals’ ownership of companies outside of the tax base and their realization of gains. This restriction balances the income earned from ownership of foreign companies (non-dollar-based). As a result, we consider this unknown error small and neglect it. Individual income represents the entire product of the economy realized by its constituent members. We can add other commodities to our model and treat them similarly to money; they increase the dimensionality of our analysis’s scalar space.

Since entropy is utility [1], we will consider our model of the economy as following Bernoulli’s logarithmic utility and be of a similar form to the Cobb–Douglas production function and that of an ideal (canonically distributed like income) gas [19] (eq 3.38),

(10) $$s=s_{0}+c_{m}R\log\frac{e}{e_{0}}+R\log\frac{m}{m_{0}}.$$

We can use Equation (10) to derive two important equations of state (see [19] (§3-4)): the ideal money equation,

(11) Pm=RT;

and a description of the internal action of an economy,

(12) $$e=c_{m}\;R\;T.$$

where *P* is the marginal value of money, *m* is the average individual income, *R* is the ideal money constant, *T* is the economic temperature (a measure of economic activity), *e* is the average exergy used by an individual, $c_{m}$ is the specific exergy capacity for a constant money supply.

**Figure 1:**
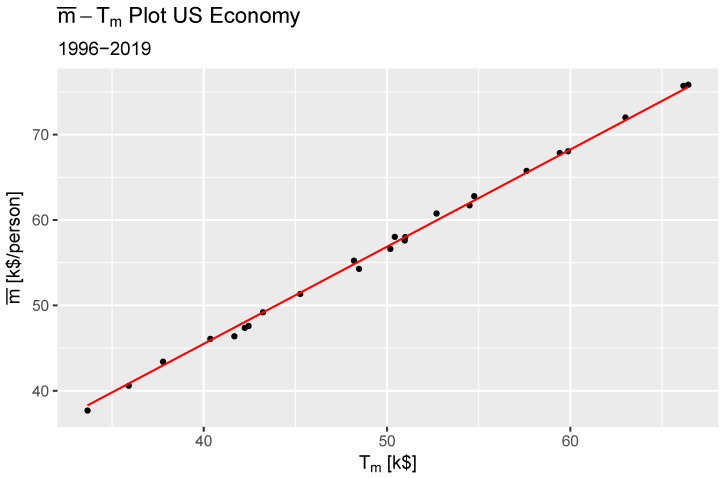
Plot of the average income versus the computed monetary temperature.

Recognizing that $\beta_{m}=P/T$ and $P=T/T_{m}$, we can express Equation (11) as

(13) $$m=R\;T_{m}.$$

which, when we perform a regression using the $T_{m}$ computed from Equation (9), we find the ideal money constant as 1.138[1/person]±0.002 with $\chi^{2}\left(1,N=23\right)=559.9,\;p<2.2\times 10^{-16}$ from a simple linear regression. Figure 1 shows the average income versus the computed monetary temperature using Equation (9). The fitted model is in red.

Ideally, we would have some measure of the economic activity, its temperature, and perform a similar regression as the ideal money constant with Equation (12). However, this is not the situation that we face. Therefore, we use *R* and subtract the contribution of money to the ensemble’s utility,

(14) $$\left(s-s_{0}\right)-R\log\frac{m}{m_{0}}=c_{m}R\log\frac{e}{e_{0}}.$$

From Equation (14), $c_{m}=0.98\pm 0.08$, with significance of $\chi^{2}\left(1,\;N=23\right)=11.93,p=2.5\times 10^{-11}$ using $s_{0}=s_{1996}$, $e_{0}=e_{1996}$, and $m_{0}=m_{1996}$ from Table A2. With parameters for Equation (10) determined, we can compute the remaining economic parameters; see Table A2.

We can test this theory against the data in an interesting manner. We recall from thermodynamics that we can define the ratio of the specific heats of an ideal gas as being

(15) $$\gamma \equiv 1+\frac{R}{c_{m}}.$$

**Figure 2:**
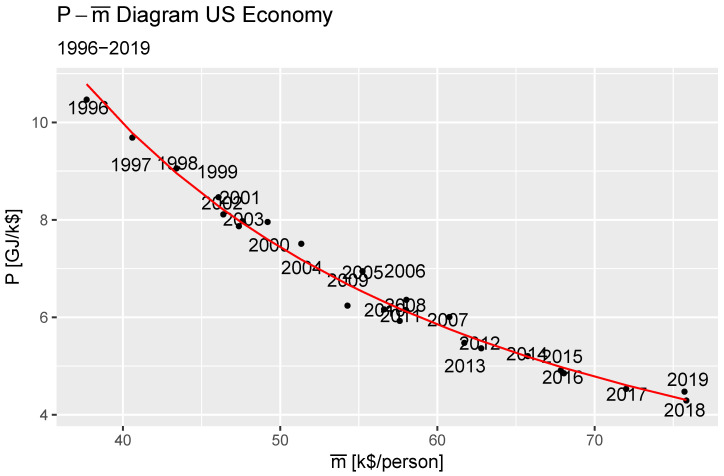
Polytropic expansion of the United States economy 1996–2019.

For our ensemble, γ=2.16. There are a number of different ways that the volume of a system can be expanded or shrunk. In general, we can model these through what is known as a polytropic process. We can describe this process as being

(16) $$P\;m^{n}=C$$

where *n* is the polytropic coefficient and *C* is a constant. We find that the US economy over the study period underwent a polytropic expansion, with n=1.31 with significance of $\chi^{2}\left(1,\;N=22\right)=-36.66,\;p<2.2\times 10^{-16}$. Figure 2 shows the data points, and the red line is the fit of Equation (16), the dollar’s demand curve, with elasticity −n.

Based on 1<n<γ and the fact that the income is generally expanding, we conclude that the economy has work extracted from it through the expansion of the money supply and that there is a net energy inflow into the economy. Where the extracted work is going remains an open question. It is entirely possible that because money creation is relegated to a small group, expanding the money supply allows them to purchase additional assets before the value has been removed from the money. These asset purchases then inflate prices. As the money diffuses farther into the economy, its value decreases until all currency has a marginally lower value.

## 4. Discussion

For this final part, we focus on the economic issue of income inequality, the nature of income distributions, and the concept of what is “fair”. For this discussion and acknowledgment of the centrality of energy in economic activity in the previous section, we represent the Hamiltonian as being the available exergy, $\hat{E}$, modifying Equation (3),

(17) $$\hat{\rho }=\frac{e^{-\beta \hat{E}}}{Z[\beta ]}.$$

There are two general notions of equality:

1. Equality of opportunity.
2. Equality of outcome.

### 4.1. Equality of Opportunity

Addressing the equality of opportunity first, for a given state, each member of the ensemble has a 1/N probability of being in a given state. Because the available energy for the ensemble is finite, the resulting maximum entropy distribution is the canonical distribution, Equation (17), where the eigenvalues of the Hamiltonian equate with the value of each state. This distribution is the exponential distribution in the continuous case. It is universal. In any case, restrictions are placed on the occupancy of the outcomes (policy entanglements), or money is taken from one and given to another, reducing the ensemble’s entropy (utility). A good way of thinking about entropy/utility is as complexity. In this context, entropy represents a quantitative measure of liberty or freedom.

As demonstrated by our society, we are allowed to own (control) property; we can accumulate wealth. Thus, the amount of wealth we have accumulated determines the amount of income (rent) we have. At its heart, rental income is a Markovian process in which each step determines the probability of the subsequent steps. The maximum entropy distribution for a continuous Markov process is the Pareto distribution, explaining why the upper portion of the income distribution is Pareto. Thus, even in this case, we still have equality of opportunity.

For a pure thermal society (a society in thermal equilibrium), the Gini coefficient will be at the low end, 0.5. For a pure epithermal society (a society where everyone is part of the Pareto distribution), if we had a Pareto exponent of 1.3, this would be a Gini of 0.625. In a society that is in the thermal equilibrium of the equality of opportunity, the minimum Gini is 0.5 and has the maximal distribution of wealth possible.

### 4.2. Equality of Outcome

If we look at the construction of the Gini Index, the line of perfect equality occurs when all the individuals in society have the same outcome. The line of equality has long been argued for as a desirable state, primarily from socialism, with a wide variety of different policies to enact the ideal state of wealth distribution. What does the mathematical conception of this ideal state look like?

First, since we are dealing with human beings living in a reality governed by the maximization of entropy, wealth is *always* canonically distributed. As previously discussed, restrictions on wealth (policy entanglements) will only lower the ensemble’s entropy. We cannot overlook the fact that the ensemble’s entropy always seeks its maximum subject to its constraints, even policy constraints. These structural impositions can lower the Gini coefficient. Any Gini < 0.5 has such policy entanglements; there will always be some level of inequality regardless of the policy restrictions.

Let us then ask if there is a theoretical situation or condition that could lead to the ideal of perfect equality. Fortunately for us, there is such a theoretical limit. Because there are quantum effects in our interactions, there is a theoretical state called a Bose–Einstein condensate (The members of a Bose–Einstein condensates can each occupy the same state (outcome). The Bose–Einstein statistics are usually derived from the Grand Canonical ensemble as a direct result because these ensembles exhibit symmetric behavior through permutation, ${\hat{P}}_{ij}|\psi \rangle =|\psi \rangle$, where ${\hat{P}}_{ij}$ is the permutation operator and |ψ〉 is a pure strategy. If the permutation is antisymmetric, ${\hat{P}}_{ij}|\psi \rangle =-|\psi \rangle$, then Fermi–Dirac statistics are followed. Games that would exhibit antisymmetric behavior are games where one can only win or loose and not share the same outcome, like sporting events). This condensate only occurs at very low energy states where the β of Equation (17) becomes very very large. In this situation, people will all occupy the same or very few states and have perfect equality of outcome. The unfortunate consequence of this is that there is little, if any, economic activity. Remember, *T* is a measure of economic activity; the hotter the temperature, the more activity there is. Conversely, if *T* is lower, the economy is colder with less activity; as β=1/T, a smaller *T* is a larger β.

Thus, theoretically and in practice, any policy that shifts from equality of opportunity to equality of outcome always reduces economic activity, and people at the societal level are always slightly worse off in proportion to the degree of the restriction. Furthermore, such outcomes only occur at extremely low levels of economic activity, e.g., in hunter-gatherer societies where there is minimal variation in roles (See the earlier discussion on the division of labor). If it is claimed that such equality can be achieved without extremely low economic activity, this would be contrary to the second law of thermodynamics. As Arthur Eddington famously said,

The law that entropy always increases holds, I think, the supreme position among the laws of Nature. If someone points out to you that your pet theory of the universe is in disagreement with Maxwell’s equations—then so much the worse for Maxwell’s equations. If it is found to be contradicted by observation—well, these experimentalists do bungle things sometimes. But if your theory is found to be against the Second Law of Thermodynamics, I can give you no hope; there is nothing for it to collapse in deepest humiliation [25].

## 5. Conclusions

By using the statistical economic framework, we have gained insights into both individual human choice and collective human action. The canonical ensemble has proved to be a handy and powerful tool for simplifying and clarifying our understanding of our interactions and our behavior. Hopefully, the two empirical examples provided show, in sufficient detail, how to formally use statistical economics as an analytical framework and how to think about and approach ensembles. We have even been able to resolve a centuries-long debate about income inequality just by studying the canonical distribution with a simple thought experiment and study of it.

This paper will hopefully prompt a more critical look into statistical economics, one that is capable of a substantive critique. Theories do not become better without being challenged. That is why this paper is a challenge to rethink our approach to economics.

## Figures and Tables

**Table 1:** The reformatted results of List and Haigh [17].

Game	Occupancy	Basis	Cohort
*A*	15, 17	|1〉, |2〉	Student
*A*	8, 19	|1〉, |2〉	Trader
*B*	8, 24	|3〉, |4〉	Student
*B*	3, 24	|3〉, |4〉	Trader
AB	4, 13, 3, 10	|1,3〉, |1,4〉, |2,3〉, |2,4〉	Student
AB	8, 7, 9, 30	|1,3〉, |1,4〉, |2,3〉, |2,4〉	Trader

**Table 2:** Ensemble specific parameters calculated from List and Haigh [17].

Game	Cohort	*N*	〈s〉	〈e〉	$\beta [{\$}^{-1}]$
*A*	Student	32	0.691	$7.13	−0.500
*A*	Trader	27	0.608	$7.18	−3.46
*B*	Student	32	0.562	$1.94	−4.39
*B*	Trader	27	0.349	$1.97	−8.32
AB	Student	30	1.23	$4.53	
AB	Trader	54	1.17	$4.55	−8.56
AB	Canonical	54	1.14	$4.56	−8.56

## Data Availability

The data and all computer code are available publicly on GitHub: 1. For the US macroeconomic analysis see, https://github.com/crabel99/US-Entropic-Analysis, accessed on 26 February 2025. 2. For the analysis of the List and Haigh data see, https://github.com/crabel99/Allais-Analysis, accessed on 26 February 2025.

## Abbreviations

The following abbreviations are used in this manuscript:

AGI	Adjusted Gross Income
API	Application Programming Interface
EIA	Energy Information Agency
EU	Expected Utility Hypothesis
HMC	Hamiltonian Monte Carlo
IRS	Internal Revenue Service
LLC	Limited Liability Company
NUTS	No U-Turn Sampling
QGT	Quantum Game Theory
SEU	Subjective Expected Utility
US	United States
vNM	von Neumann—Morgenstern

## Appendix A

**Table A1:** Regression coefficients of the United States income tax distribution for the mixed exponential–Pareto distribution for 1996–2019.

Year	$T^{⋆}\left[\$\right]$	$m_{0}\left[\$\right]$	α	*Z*
1996	33,424.95	120,921.4	1.197789	32,783.57
1997	35,533.71	127,040.7	1.137222	34,999.70
1998	42,384.76	118,780.6	1.272331	40,485.83
1999	39,053.37	135,461.6	1.105257	38,521.05
2000	39,552.88	129,135.1	1.195418	38,577.32
2001	40,889.55	131,838.8	1.309683	39,434.20
2002	41,338.96	133,736.0	1.363438	39,692.01
2003	39,862.09	123,672.2	1.346527	38,181.91
2004	41,206.12	126,851.5	1.273806	39,714.94
2005	42,661.85	128,592.3	1.227064	41,236.77
2006	44,281.24	130,389.2	1.223103	42,733.78
2007	45,653.34	135,186.9	1.207421	44,153.72
2008	46,083.54	142,474.8	1.251603	44,528.59
2009	45,865.92	146,023.1	1.332454	44,095.15
2010	46,247.19	147,527.9	1.277674	44,690.98
2011	47,286.28	144,684.1	1.302614	45,422.53
2012	48,434.39	144,688.4	1.231663	46,758.26
2013	50,636.73	149,014.3	1.322918	48,356.20
2014	52,275.46	150,729.5	1.289789	49,987.84
2015	53,713.46	156,673.1	1.278110	51,487.21
2016	55,070.56	159,914.6	1.310235	52,585.58
2017	57,066.31	164,395.7	1.282268	54,608.08
2018	59,700.10	170,770.0	1.279972	57,100.77
2019	61,345.95	169,178.0	1.321514	58,167.35

**Table A2:** Economic variables from the canonical production function that was derived from the income distribution and estimates of exergy usage in the United States for 1996–2019.

Year	*P* [GJ/k$]	*m* [k$/person]	*N* [people]	*e* [GJ/person]	*T* [GJ]	*s* [1/person]
1996	10.467146	37.68948	120,351,210	392.1858	352.3444	11.51733
1997	9.687384	40.59687	122,421,993	387.1879	347.8541	11.59368
1998	9.056673	43.40742	124,770,661	380.8353	342.1469	11.75771
1999	8.463946	46.07878	127,075,147	380.2339	341.6066	11.70065
2000	7.956918	49.20155	129,373,502	382.9225	344.0221	11.69841
2001	7.869127	47.37317	130,255,240	369.9594	332.3759	11.70397
2002	8.111589	46.38492	130,076,442	376.23	338.0094	11.70206
2003	7.973448	47.5919	130,423,630	376.7065	338.4376	11.67136
2004	7.508802	51.34242	132,226,043	378.2977	339.8671	11.72381
2005	6.945434	55.23813	134,372,680	372.5771	334.7277	11.77325
2006	6.358212	58.02852	138,394,756	356.8393	320.5886	11.81362
2007	6.004935	60.76228	142,978,808	352.2324	316.4497	11.84845
2008	6.149047	58.0051	142,450,569	349.0134	313.5578	11.8414
2009	6.238692	54.28291	140,494,129	336.5923	302.3985	11.814
2010	6.162976	56.61016	142,892,054	344.2103	309.2426	11.83573
2011	5.925432	57.60562	145,370,240	336.1274	301.9808	11.85409
2012	5.363138	62.7905	144,928,473	326.8532	293.6487	11.89954
2013	5.474273	61.71394	147,351,299	332.075	298.3401	11.91875
2014	5.202737	65.75103	148,606,578	333.6133	299.7221	11.96088
2015	4.907715	67.84563	150,493,262	324.588	291.6137	11.9909
2016	4.850748	68.04946	150,272,156	323.2772	290.436	12.00673
2017	4.530385	72.00567	152,903,232	317.7014	285.4267	12.05083
2018	4.473874	75.71772	153,774,296	329.4796	296.0083	12.09697
2019	4.291296	75.83724	157,796,805	317.3104	285.0754	12.11267
